# Use of the PREPARE (PREhabilitation, Physical Activity and exeRcisE) program to improve outcomes after lumbar fusion surgery for severe low back pain: a study protocol of a person-centred randomised controlled trial

**DOI:** 10.1186/s12891-016-1203-8

**Published:** 2016-08-18

**Authors:** Hanna Lotzke, Max Jakobsson, Helena Brisby, Annelie Gutke, Olle Hägg, Rob Smeets, Marlies den Hollander, Lars-Eric Olsson, Mari Lundberg

**Affiliations:** 1 Department of Orthopaedics, Institute of Clinical Science, Sahlgrenska Academy, University of Gothenburg, Gothenburg, Sweden; 2 Spine Center Göteborg, Västra Frölunda, Sweden; 3 District Department North, Division of Rehabilitation, Borås Stad, Borås, Sweden; 4 Division of Physiotherapy, Department of Health and Rehabilitation, Institute of Neuroscience and Physiology, University of Gothenburg, Gothenburg, Sweden; 5 Department of Rehabilitation Medicine, Maastricht University, Maastricht, The Netherlands; 6 Libra Rehabilitation and Audiology, Eindhoven/Weert, The Netherlands; 7 Department of Rehabilitation Medicine, Maastricht University, Medical Centre, Maastricht, The Netherlands; 8 Department of Clinical Psychological Science, Maastricht University, Maastricht, The Netherlands; 9 Division of Physiotherapy, Department of Neurobiology, Care Sciences and Sociology, Karolinska Institutet, Stockholm, Sweden; 10 Department of Orthopaedics, Sahlgrenska University Hospital, Gothenburg, Sweden; 11 Institute of Health and Care Sciences, Sahlgrenska Academy, University of Gothenburg, Gothenburg, Sweden; 12 Gothenburg Centre for Person-Centred Care (GPCC), University of Gothenburg, Gothenburg, Sweden; 13 Physiotherapy Department Spine Center AB, 430 21 Västra Frölunda, Sweden

**Keywords:** Prehabilitation, Rehabilitation, Spinal fusion surgery, Physiotherapy, Cognitive behavioural approach, Person-centred, Chronic low back pain, Physical activity

## Abstract

**Background:**

Following lumbar fusion surgery, a successful outcome is empirically linked to effective rehabilitation. While rehabilitation is typically postoperative, the phase before surgery – termed *prehabilitation* – is reportedly an ideal time to prepare the patient. There are presently no guidelines for prehabilitation before lumbar fusion surgery. Physical activity has well-known health benefits, and staying physically active despite pain is a major principle in non-pharmacological chronic low back pain treatment. Psychological factors such as fear of movement, pain catastrophizing and low self-efficacy are known to be barriers to staying active. No studies have investigated prehabilitation protocols that promote physical activity and target psychological risk factors before lumbar fusion surgery. The aim of our proposed randomised controlled trial is to investigate whether patients who undergo lumbar fusion surgery for degenerative disc disease experience better functioning with a physiotherapeutic prehabilitation program (PREPARE) based on a cognitive behavioural approach compared to conventional care.

**Methods/Design:**

We will recruit 110 patients between 18–70 years of age with degenerative disc disease who are waiting for lumbar fusion surgery. These patients will be randomly assigned to receive either PREPARE or conventional care. PREPARE uses a person-centred perspective and focuses on promoting physical activity and targeting psychological risk factors before surgery. The primary outcome will be disability measured using the Oswestry Disability Index 2.0. Secondary outcomes will include functioning (patient-reported and performance-based), physical activity (accelerometer), health-related quality of life, back and leg pain intensity, pain catastrophizing, kinesiophobia, self-efficacy, depression, anxiety, satisfaction with treatment results and health economic factors. Data will be collected at baseline (preoperatively) after the intervention (preoperatively), 3 and 8 weeks, 3, 6, 12, 24 and 60 months postoperatively.

**Discussion:**

We hypothesise that the focus on promoting physical activity and targeting psychological risk factors before surgery will decrease disability and help the patients to be more active despite pain both before and after surgery. We will use a combination of outcome measures both patient-reported and performance-based, as well as accelerometer data. This will provide a more comprehensive picture of the patient’s functioning than just patient-reported outcomes alone.

**Trial registration:**

Current Controlled Trials ISCRTN17115599, Retrospectively Registered 18 May 2015.

## Background

Lumbar degenerative disc disease (DDD) is a subgroup of chronic low back pain (CLBP). When pain and disability are severe, lumbar fusion surgery can be an option [1]. The frequency of lumbar spinal fusion surgery is increasing worldwide [2, 3]. In 2008, DDD was the most common diagnosis prior to spinal fusion surgery in the USA [4]. In 2013, the Swedish Spine Register recorded 8144 patients with degenerative lumbar spine disorders, of which approximately 10 % had a primary diagnosis of DDD or isthmic spondylolisthesis and underwent lumbar fusion surgery [5]. The DDD group is an interesting study group for several reasons. They are relatively young [5], and their lack of functioning has hence a large impact on their life and on society. Clinical experience dictates that most patients choose fusion surgery to enable them to continue to work and to live an active life.

While rehabilitation traditionally starts postoperatively, the phase before surgery – termed *prehabilitation* – is suggested to be an ideal time to prepare patients for an optimal outcome of surgery [6, 7]. A small number of studies of low-quality suggest that there is no evidence for the effectiveness of physiotherapy interventions before and after lumbar fusion, and hence best practice remains unclear [8]. We lack guidelines for the pre and post-operative phase for this patient group [9, 10], but there are national and international guidelines for conservative or non-pharmacological treatment for patients with CLBP. These guidelines incorporate major treatment principles, such as to stay active despite pain along with supervised exercise therapy, cognitive behavioural therapy and multidisciplinary treatment [11]. There is no reason to believe that patients with DDD would not benefit from the same treatment principles.

The benefits of staying or becoming more physically active are manifold. The major underlying rationale is the effect of physical activity on general health [12, 13]. On a global level physical inactivity is one of the global burdens, leading to premature death and non-communicable diseases [13]. It is hence important to promote physical activity in all conditions, patients with CLBP alike. Patients with DDD who choose to undergo surgery have experienced pain for several years, and 52 % of them reported that they are able to walk less than one kilometre [3]. However, there are presently little data regarding physical activity levels in this patient group. In addition to its general health benefits, physical activity has an analgesic effect that likely involves several partially overlapping mechanisms [14, 15]. The hypoalgesic effect of exercise on pain is usually reduced in patients with “more general” chronic pain conditions [14]. However, the exercise-induced hypoalgesic effects in patients with CLBP are reportedly similar to those in healthy individuals [16, 17]. It would hence be of value to promote physical activity for patients with DDD.

There are various reasons for not being physically active. One factor is the perception and attitude towards exercises [18]. Another factor is fear of movement. This factor has been identified as an important mediator in combination with catastrophizing thoughts regarding disability, disuse and depression in patients with CLBP [19]. It appeared that 70 % of the patients with CLBP who visited an orthopaedic department in Sweden for a potential fusion surgery reported kinesiophobia [20]. Even though the prevalence of kinesiophobia among patients with DDD is unknown, research suggests that the prevalence of kinesiophobia in patients with specific CLBP is similar to that in patients with non-specific CLBP [21]. Furthermore, pre-surgical psychological status such as pain catastrophizing [22], negative outcome expectations [23] and fear-avoidance beliefs [24] have been found to be significant predictors of pain and function up to 2 years after lumbar surgery. Hence, we hypothesise that the majority of persons who are waiting to undergo surgery for DDD suffers from a high degree of pain, kinesiophobia, catastrophizing and low self-efficacy – and all of these factors may contribute to a low physical activity level. Hence, an intervention using a cognitive behavioural approach could be appropriate to target these risk factors. Given the multidimensional nature of pain, rehabilitation programs based on cognitive-behavioural therapies (CBT) have been specifically recommended for patients with CLBP and are often used in addition to other therapies such as exercise [25].

Although the importance of post-lumbar surgery rehabilitation programs targeting the above-mentioned psychological factors have been emphasised [26–28], so far only two programs have yielded successful outcomes following lumbar fusion surgery [28, 29]. Abbott et al. compared postoperative psychomotor therapy with exercise therapy in a randomised controlled trial (RCT) that included 107 patients undergoing lumbar fusion surgery for spinal stenosis, spondylosis, degenerative/isthmic spondylolisthesis or DDD. The psychomotor therapy program combined motor control exercises for lumbopelvic stabilisation with a cognitive behavioural intervention immediately after surgery. This treatment was more effective compared to exercise therapy in reducing disability and fear-avoidance beliefs and in increasing self-efficacy and outcome expectancy at 2 and 3 years post-surgery [28]. Another recent RCT compared a rehabilitation program including active exercise alone with a treatment combining active exercise with the management of catastrophizing and fear of movement among 130 patients undergoing lumbar fusion surgery for degenerative spondylolisthesis and/or lumbar spinal stenosis. The combined rehabilitation program showed superior disability reduction measured by the Oswestry Disability Index (ODI) at the 1-year follow-up [29].

These prior investigations have been performed after lumbar fusion surgery. However, some authors hypothesise that further optimisation of lumbar fusion surgery outcomes may be possible by initiating an intervention *before* surgery [26, 30]. Nielsen et al. found their intervention to be superior to conventional care with regards to postoperative functionality assessed by the Roland-Morris Disability Questionnaire, faster recovery and shorter hospital stay. The content of the intervention was an individualised preoperative training program that focused on improvement of muscular back strength as well as cardiovascular conditioning before spinal fusion surgery [26].

To our knowledge, at the start of our trial (2014), no prior study had assessed the use of a prehabilitation program with individual sessions to target psychological risk factors – such as fear-avoidance beliefs and low self-efficacy – to maintain or increase physical activity both before and after surgery. Therefore, we initiated an RCT to evaluate a physiotherapeutic prehabilitation program called PREPARE (*Pre*habilitation, *P*hysical *A*ctivity and exe*R*cis*E*).

### Aim of the study

The overall aim of the trial is to investigate whether PREPARE – a physiotherapeutic prehabilitation program based on a cognitive behavioural approach – will improve functioning after lumbar fusion surgery in patients with DDD compared to conventional care.

### Hypotheses

1. Patients who receive PREPARE – a physiotherapeutic person-centred prehabilitation program based on a cognitive behavioural approach – before spinal fusion surgery will experience decreased disability levels, improved functioning, increased physical activity level, better health-related quality of life, decreased leg and back pain intensity, less pain catastrophizing, less pain-related fear, increased self-efficacy to exercise and less depressed mood after surgery compared to patients who receive conventional care. We further hypothesise that the between-groups difference will be largest at 6 months after surgery.
2. Compared to those in the control group, patients who receive PREPARE will show a more rapid increase in their physical activity level measured objectively with an accelerometer at 3 and 6 months after surgery.
3. From a societal perspective, PREPARE will be more cost-effective and show higher cost-utilities compared to conventional care at one year after surgery.

## Methods/Design

### Trial design

A prospective RCT will be performed at a private spine clinic in Gothenburg, Sweden. Participants will be randomised to one of the two arms of the trial.

### Theoretical Framework

The PREPARE program combines different philosophical standpoints, theoretical models and treatment principles.

Pain is defined as *“an unpleasant and emotional experience associated with actual or potential tissue damage, or described in terms of such damage”* [31], and is hence to be considered a subjective phenomenon. As PREPARE rests on the assumption that pain is subjective, we will apply the principles of person-centred care (PCC). In PCC, emphasis is placed on recognising the patient as a human being with reason, will, feelings, and need, and on engaging the patient as an active partner in his/her care and treatment [32]. The most important parts in PCC is the partnership between the patient and the therapist and to formulate a health plan together with the patient which includes both short-and long-term goals along with the actions needed to reach each goal.

**Fig. 1:**
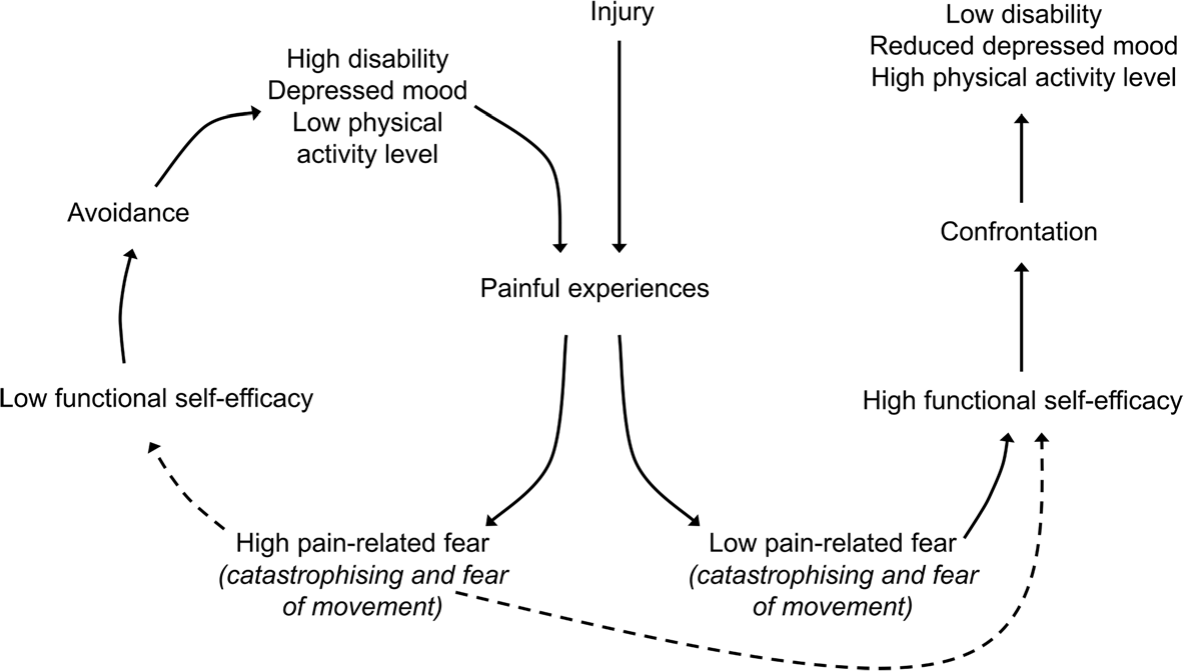
The fear-avoidance model with the mediating role of self-efficacy

Modern pain research has led to a shift in treatment strategies, from pain reduction to pain management with a focus on teaching patients to manage their thoughts (cognitive component) and feelings (affective component), which are both pain-related components. PREPARE will target fear-avoidance beliefs, as well as self-efficacy, which are regarded as important for a person’s ability to stay active despite pain [33]. For patients of working age with LBP, evidence suggests that treatment should include cognitive behavioural techniques combined with physical activity [34]. Self-efficacy is a concept that explains a person’s confidence in their ability to perform a specific activity [35]. In patients with LBP, both self-efficacy and fear-avoidance beliefs are possible predictors of disability [33, 36]. However, since it remains unclear how these two constructs differ in predicting disability, it is suggested that therapists measure and consider both constructs when treating patients with LBP [36, 37]. To achieve this, the physiotherapist will use a combination of techniques based on a cognitive behavioural approach [38]. Figure 1 presents an overview of the fear-avoidance model including the mediating role of self-efficacy.

### Participants

We will recruit a total of 110 patients of 18–70 years of age, who have major complaints of DDD: CLBP of motion-elicited type degenerative changes of 1-3 segments of the lumbar spine, reproducible pain in clinical examination assessed to originate in the same segment as demonstrating degenerative changes, and on the waiting list for lumbar fusion surgery. Patients may have additional minor radiating symptoms with or without simultaneous surgical procedure for disc herniation, foraminal decompression, or isthmic spondylolisthesis. The trial will exclude patients who have undergone previous decompression surgery for spinal stenosis or who have spinal malignancy, dominating radiculopathy, a confirmed neurological disorder or rheumatic disorder, deformities in the thoracolumbar spine (e.g. idiopathic scoliosis), or a poor understanding of the Swedish language.

### Recruitment and randomization

Trial participants will be recruited from two private spine clinics and one university hospital. Patients will be clinically examined by an orthopaedic surgeon, who will make a medical diagnosis based on the clinical and radiological findings. Patients who are diagnosed with DDD will be informed that lumbar fusion surgery is a treatment option. When a patient agrees to undergo surgery and is placed on the waiting list, the hospital and the spine clinics coordinators will inform the physiotherapist (*PT-prepare*) who will deliver the trial intervention. Then the *PT-prepare* will call the patient to inform him/her of the trial and invite him/her to participate.

Patients interested in participating will meet with an independent observer at baseline, 8–12 weeks before surgery at one of the private spine clinics. The independent observer will once again provide the patient with information about the trial. If the patient agrees to participate, he/she will sign an informed consent form provided by the independent observer. The independent observer will then guide the participant through the functional capacity testing and provide him/her with the patient-reported outcome measures and the accelerometer. After this, the independent observer will give the participant a sealed envelope containing the group allocation.

The participant will be randomly allocated to Group A or Group B using a computerised random list with no restrictions, arranged by an independent statistician. The project leader – who will not take part in the intervention and outcome assessments – will be responsible for arranging sequentially numbered, opaque, sealed envelopes. To conceal the group allocation, the participant will not open this envelope in the presence of the independent observer. The allocation list will be stored in a locked fire-proof cupboard at the university laboratory, in accordance with the principles of the ethical approval.

### Blinding

The independent observer who is responsible for all measurements will be blinded to the treatment allocation. The trial participants will be aware of the treatment that they receive, but will not know the content of the treatment in the control group. The *PT-Prepare* will not be blinded since she is delivering the treatment for Group A.

### Intervention

**Fig. 2:**
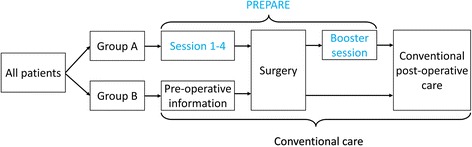
Overview of the intervention groups

Group A (*n* = 55) will be treated using the PREPARE protocol. Group B (*n* = 55) will receive conventional care, including general advice to stay active before surgery. Figure 2 presents an overview of the intervention groups.

#### Group A

**Fig. 3:**
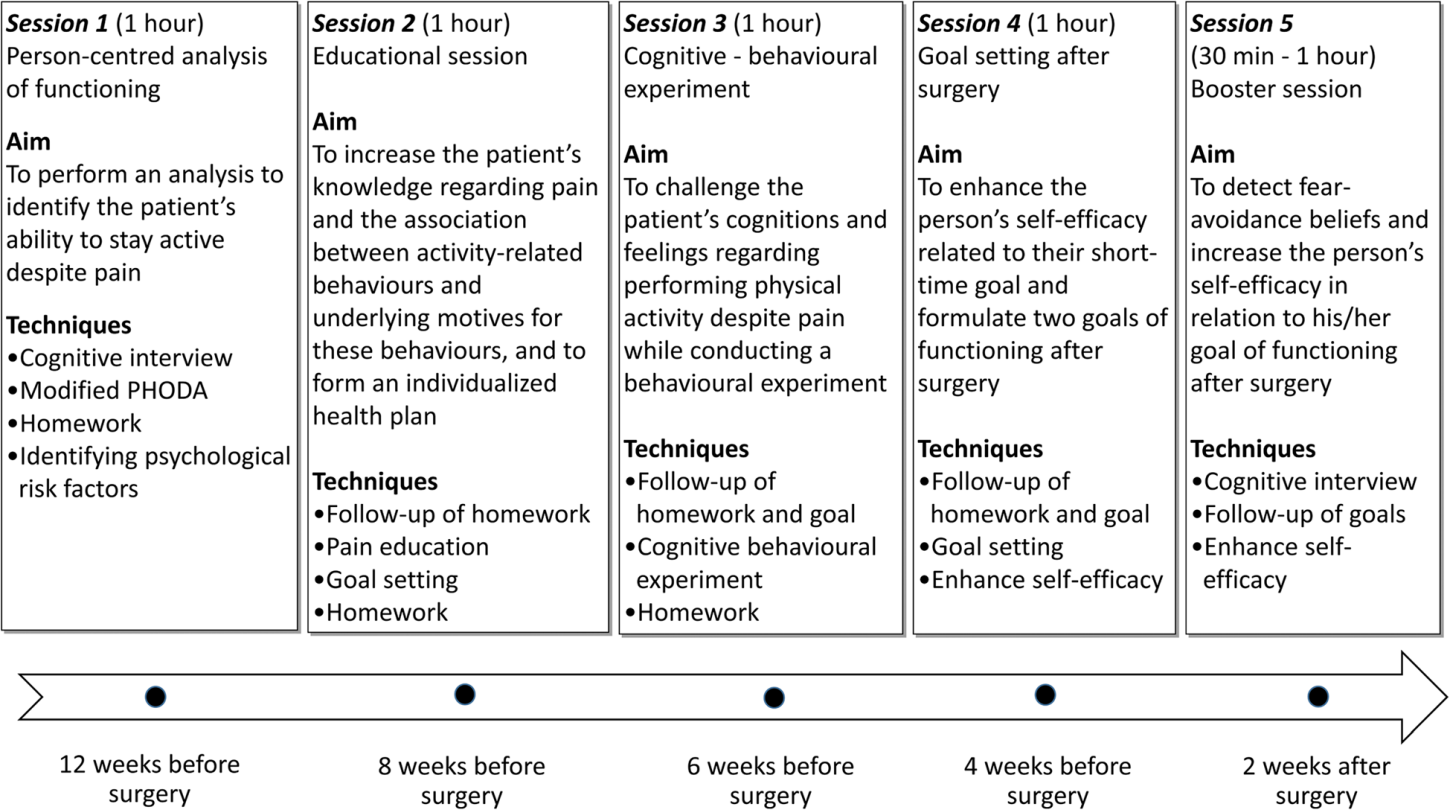
Overview of the intervention

The PREPARE protocol includes four individually tailored treatment sessions before surgery, and one booster session over the telephone two weeks after surgery. Depending on the patient’s schedule, PREPARE typically starts about 8 to 12 weeks *before* surgery. Each treatment session lasts for about one hour, and the post-surgery booster session lasts about 30 min. The intervention will be delivered by a physiotherapist with 1.5 years of CBT training. Figure 3 presents a schematic overview of the intervention.

##### Session 1 – Person-centred analysis of functioning

The aim of session 1 is to perform an analysis to identify the patient’s ability to stay active despite pain.

- *Cognitive interview with a Socratic approach* The patient participates in a cognitive interview aimed at capturing the patient’s cognitions and feelings relating to staying active despite pain. To direct the interview’s focus towards functioning rather than pain, the *PT-prepare* will discuss with the patient what kinds of physical activities he/she wishes to be able to perform after surgery.
- *Modified PHODA – analysis of activity behaviour* To capture the patient’s cognitions and feelings regarding activity behaviours related to physical activities and the underlying motives of these behaviours, a modified version of the Photograph Series of Daily Activities (PHODA) will be used (described below) [39], which is extended to include additional photographs of leisure-based physical activities.
- *Homework* Homework will be assigned with a two-fold aim: to help the patient learn to be aware of cognitions, feelings, behaviour, and body sensations related to a physical activity; and to increase or maintain his/her physical activity level. The patient will first make a list of physical activities that he/she would like to do, but has stopped performing or has been performing less often or less intensely due to his/her back problem. The patient will then select one of these physical activities, and monitor his/her cognitions, feelings, behaviours, and body sensations related to that physical activity as homework until session 2.
- *Identifying psychological risk factors* Questionnaires will be used to identify psychological risk factors for low functioning. Before session 2, the patient will complete the following questionnaires: Pain Catastrophizing Scale, Tampa Scale for Kinesiophobia and Self-efficacy for Exercise (described below).

##### Session 2 – Educational session

The aim of session 2 is to increase the patient’s knowledge regarding pain and the association between activity-related behaviours and underlying motives for these behaviours and form an individualised health plan.

- *Follow-up of homework* The session will start with a discussion of the patient’s cognitions, feelings, behaviours and body sensations related to the physical activity that he/she has monitored as homework.
- *Pain education* The patient will participate in an educational session about acute and chronic pain [40]. The theoretical underlying model for this pain education is the fear avoidance model presented by Vlaeyen et al. [19] and modified by Woby et al. [33]. The pain education session will be conducted as a dialogue between the *PT-prepare* and the patient, with the *PT-prepare* considering the patient’s thoughts, beliefs and knowledge as well as what he/she wants to know about staying active despite pain in relation to his/her pain condition. The session will be guided by the patient’s responses to the questionnaires completed after session 1, as well as the information from the patient’s homework assignment. This will help the *PT-prepare* to form a health plan together with the patient and decide upon a person-centred goal in relation to the underlying theoretical models of the intervention.
- *Goal setting – short-term goal* The patient will choose a person-centred short-term goal related to functioning that he/she will aspire to achieve before surgery. This short-term goal should be specific, measurable, achievable, realistic and time-targeted (SMART-goal) [41] and should involve a physical activity that is important to the patient. For example, a short-term goal could be to “*go for a walk in the nearby forest for 30 min every day*”. The patient will be encouraged to work towards the goal in gradual steps to enhance his/her self-efficacy related to that physical activity.
- *Homework* The homework assignment will have a three-fold aim: for the patient to learn to be aware of cognitions, feelings, behaviours and body sensations related to a physical activity; for the patient to increase his/her physical activity level, and finally to help the patient reach his/her short-time goal before surgery. The patient will choose this physical activity from the list of physical activities that he/she established during session 1.

##### Session 3 – Cognitive behavioural experiment

The aim of session 3 is to challenge the patient’s cognitions and feelings regarding performing physical activity despite pain while conducting a behavioural experiment.

- *Follow-up of homework and short-term goal* This session will start with a discussion of the patient’s progress towards their short-term goal, as well as their cognitions, feelings and behaviours related to the physical activity that he/she has performed as homework.
- *Cognitive behavioural experiment* Under the supervision of the *PT-Prepare*, the patient will perform a behavioural experiment aiming to enhance inhibitory learning by testing and violating negative expectations that the patient might have regarding a physical activity. For the behavioural experiment in this session, the patient will select one physical activity from the list of physical activities established during session 1. Before the behavioural experiment, the patient will be asked about what cognitions he/she has about the activity and what he/she expects will happen when performing that physical activity. After performing the physical activity, the validity of the patient’s thoughts and the patient’s level of self-efficacy will be discussed in terms of both short- and long-term consequences.
- *Homework* The three aims of the homework will be for the patient to learn to be aware of cognitions, feelings, behaviours and body sensations related to a physical activity, to increase his/her physical activity level, and to help the patient reach his/her short-term goal before surgery. The homework will be either the same physical activity selected during session 2, or a new physical activity from the list of physical activities established during session 1.

##### Session 4 – Goal setting after surgery

The aim of session 4 is to enhance the patient’s self-efficacy related to their short-term goal and to formulate two functioning-related goals to be reached at 4 and 8 weeks after surgery.

- *Follow-up of homework and short-term goal* This session will start with a discussion of the patient’s progress of his/her short-term goal and of the patient’s cognitions, feelings and behaviours related to the physical activity that he/she has performed as homework.
- *Goal setting – 4 and 8 weeks after surgery* The patient will choose two functioning related goals to reach at 4 and 8 weeks after surgery, which should involve a physical activity that is important to the patient. These goals will be set as SMART-goals [41].
- *Enhance self-efficacy related to short-time goal* The patient will be encouraged to continue working towards his/her short-term goal, and will be advised to take gradual steps towards enhancing his/her self-efficacy related to that goal. If the patient has already reached the short-term goal, the goal will be modified by increasing the intensity, duration, or frequency of the physical activity or by choosing another physical activity that the patient considers to be important.

##### Session 5 – Booster session

The aim of session 5 is to detect fear-avoidance beliefs and to increase the patient’s self-efficacy in relation to his functioning goals for 4 and 8 weeks after surgery. This session acts as a booster session that will be held over the phone at two weeks after surgery.

- *Cognitive interview with a Socratic approach* The patient will participate in a cognitive interview that aims to capture his/her cognitions and feelings regarding physical activities – particularly activities of daily life (ADL). The *PT-prepare* will identify tendencies towards fear-avoidance beliefs and encourage the patient to stay active.
- *Follow-up of goals 4 and 8 weeks after surgery* The patient’s goals will be discussed and will be adjusted with regards to duration, intensity, or frequency in accordance with the patient’s current medical status.
- *Enhance self-efficacy related to goals 4 and 8 weeks after surgery* The patient will be encouraged to continue with his/her progress towards the goals, and will be advised to take gradual steps towards enhancing his self-efficacy related to that physical activity.

#### Group B

Patients in Group B will receive a conventional care intervention. Before surgery, these patients will be encouraged to contact a physiotherapist at one of the private spine clinics to schedule an informational session. There, they will be provided with basic information about the upcoming surgery, and told about the types of core exercises that will be introduced at the ward post-surgery. The physiotherapist will encourage the patients to stay active and to start performing the recommended exercises prior to their surgery.

#### Common features for Group A and B after surgery

The aim of this trial is to evaluate the effects of PREPARE rather than the postoperative treatment of the patients in Group A. Both groups will receive the same post-surgical treatment, aside from the booster session for Group A. After surgery, the patients will meet with a physiotherapist at the ward, and participants in both groups will receive the same information regarding post-operative rehabilitation. At the ward, both groups will be instructed to perform the same home exercise program comprising core stabilisation exercises for the first four weeks after surgery. All patients will be advised to stay active on a daily basis, and to contact a physiotherapist from a rehabilitation centre or physiotherapy clinic in their local area at four weeks after the surgery to continue their rehabilitation. The content of this rehabilitation will be determined by the treating physiotherapist and will not be controlled for.

### Outcome measures

#### Primary outcome measure

- Disability will be measured using the revised version (version 2.0) of the Oswestry Disability Index (ODI). In the ODI, patients rate their perceived disability on 10 items relating to pain intensity, personal care, lifting, walking, sitting, standing, sleeping, sex life, social life and traveling Each item is scored from 0–5, with 0 indicating the least amount of disability and 5 the most severe disability. The scores are summed to generate a total score ranging from 0 to 100, with 0 indicating no disability. The English version of the ODI shows high internal consistency [42] and test–retest reliability [43], as well as adequate content validity and responsiveness [44] for patients with CLBP.

#### Secondary outcome measures (effect evaluation)

- Patient-reported functioning will be measured using the Swedish version of the Patient-Specific Functional Scale (PSFS). In the PSFS, the patient lists three activities that are limited by the condition for which he/she is seeking treatment. The patient then rates their perceived difficulty in performing each of the listed activities on a scale from 0 to 10, with 0 indicating that the patient cannot perform the activity at all. The three separate scores are averaged to generate the total score The English version of the PSFS is responsive to clinically important change over time [45], and shows good test–retest reliability and strong criterion validity [46] for patients with CLBP.
- Performance-based functioning will be measured using five tests: 5-min walking (measurement of the distance the patient can walk in 5 min); 50-feet fast walking (measurement of the time it takes for a patient to walk 50 feet as fast as possible); the Timed up-and-go (measurement of the time it takes for a patient to arise from a chair, walk three meters, turn around, walk back to the chair and sit down); 1-min stair-climbing (measurement of the number of steps the patient can climb in one minute) [47, 48]; and the One-Leg Stand Test (measurement of the duration the patient can stand on one leg, with eyes open and with eyes closed) [49]. Findings support the construct validity of these tests and show that they have adequate test–retest reliability for patients with CLBP [47–49].
- A digital triaxial accelerometer (ActiGraph GT3X+; ActiGraph, Pensacola, FL, USA) will be used to assess physical activity as steps per day, time spent at different intensity levels and time spent sedentary [50, 51]. The GT3X+ measures acceleration in three planes and the raw output is “counts”. Based on the number of counts per minute, the raw output is classified into time spent at different intensities of physical activity and time spent sedentary by using appropriate cut-points in the complementary software, Actilife 6 (ActiGraph, Pensacola, FL, USA). The device will be attached with an elastic band to the patient’s right iliac crests during waking hours for a 7-day period. The patient will be instructed to remove the device while sleeping, swimming and bathing. The GT3X+ has shown high construct validity when measuring physical activity intensity levels [50] and excellent criterion validity when measuring the number of steps [51] in healthy adults.
- Health-related quality of life will be measured using the Swedish version of the European Quality of Life 5 Dimensions Questionnaire (EQ-5D). In the EQ-5D, patients rate their health on five items relating to mobility, self-care, usual activities, pain/discomfort and anxiety/depression. The patient also rates his/her general health on a visual analogue scale. The English version of the EQ-5D has acceptable test–retest reliability and findings support its construct validity for patients with chronic musculoskeletal pain [52].
- Health-related quality of life will also be measured using the Swedish version of the Short Form (36) Health Survey (SF-36). The SF-36 comprises eight scaled sections: vitality, physical functioning, bodily pain, general health perceptions, physical role functioning, emotional role functioning, social role functioning and mental health [53]. The Swedish version of the SF-36 shows high internal consistency and high discriminant validity and findings support its construct validity in a Swedish population sample [54].
- Back and leg pain intensity levels over the last week will be measured using 100-mm Visual Analogue Scales (VAS) [55]. Findings support the validity and reliability of the VAS in patients with chronic pain [56].
- Pain will also be measured using a 5-point scale with the following response options: pain-free, much better, better, unchanged, or worse. This scale shows good responsiveness for patients with CLBP undergoing lumbar fusion surgery [57].
- Pain catastrophizing will be measured using the Swedish version of the Pain Catastrophizing Scale (PCS), which includes 13 items assessing catastrophizing thoughts about pain. The PCS has shown adequate internal consistency and high construct validity in a Swedish population sample [58].
- Kinesiophobia will be subjectively rated using the Swedish version of the Tampa Scale of Kinesiophobia (TSK-SV). The TSK comprises 17 items assessing the subjective kinesiophobia rating. The TSK-SV has shown high test–retest reliability and internal consistency, and has support for its face, content and construct validity in patients with CLBP [59].
- Self-efficacy related to exercise will be measured using the Swedish version of the Self-Efficacy for Exercise scale (SEE-SV). On the SEE-SV, the patient rates his/her confidence that he/she could exercise three times per week (20 min each session) under nine different conditions – for example, “if you experienced pain while you exercised” or “if you felt tired”. The SEE-SV shows substantial test–retest reliability and satisfactory internal consistency and content validity for older adults [60].
- Anxiety and depression will be assessed using the Swedish version of the Hospital Anxiety and Depression Scale (HADS) [61]. In a Swedish population sample, the HADS showed moderate internal consistency and high construct validity [62].
- Satisfaction with treatment results will be measured using a 3-point scale with the following response options: satisfied, uncertain, or dissatisfied.
- PHODA is an instrument that is conventionally used to determine the perceived harmfulness of daily activities in patients with LBP [39]. In this trial, we will use a modified version of PHODA to capture the patients’ beliefs and attitudes regarding activity behaviour, rather than the expected harmfulness. The original PHODA is extended with photos of leisure-based activities that the patient is also asked to rate in the above-described manner. The modified PHODA will not be used to measure outcomes of the present trial, but will rather be employed as a tool to supply the *PT-prepare* with information about the patient’s activity behaviour in relation to the pictured physical activities and the motives underlying the activity behaviour.

#### Secondary outcome measures (economic evaluation)

- Healthcare costs and to value production loss will be measured using a self-reported custom-made health economic questionnaire.

### Data collection

**Fig. 4:**
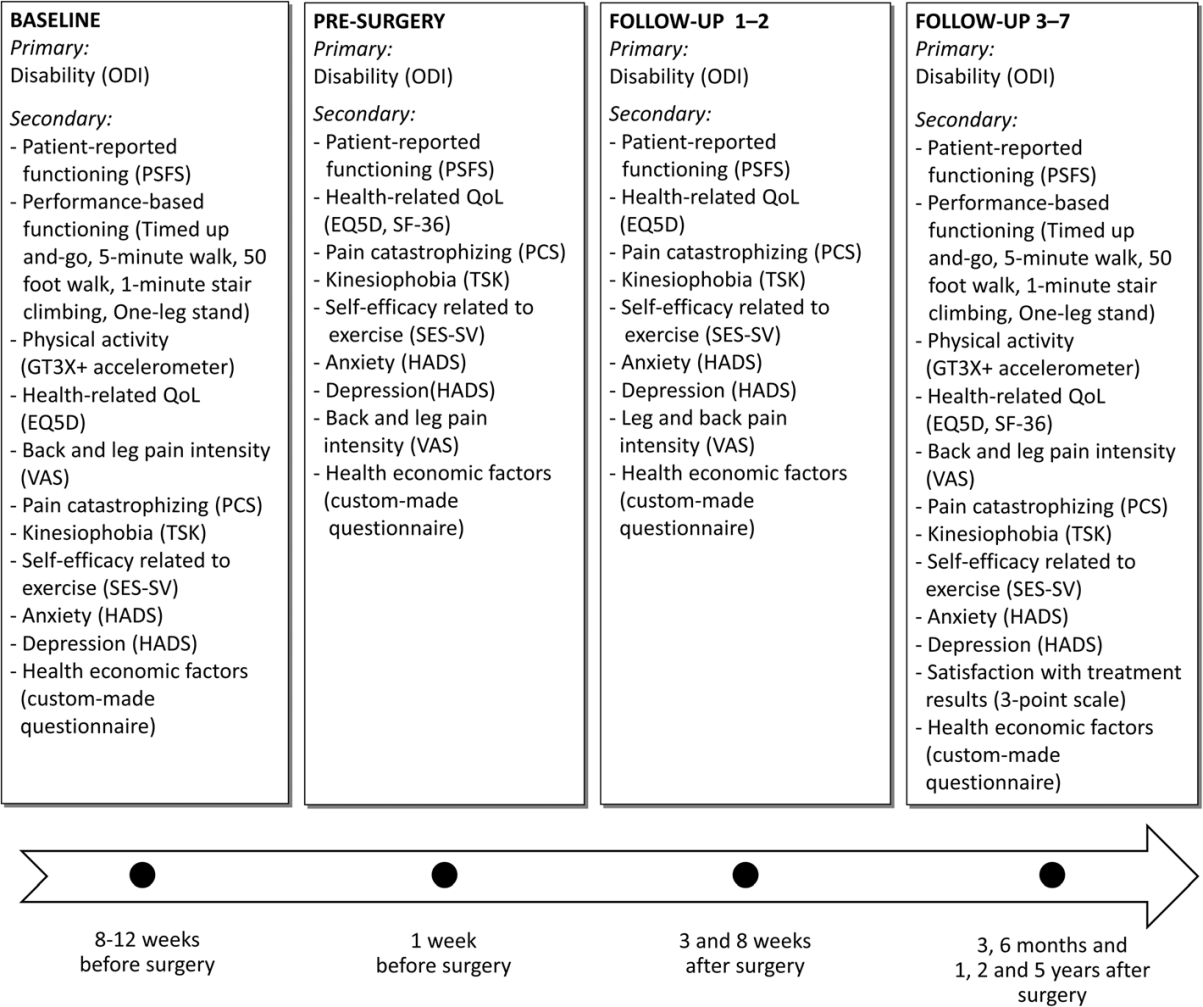
Overview of the baseline and follow-up sessions. EQ-5D, EuroQol 5 Dimensions Questionnaire; HADS, Hospital Anxiety and Depression Scale; ODI, Oswestry Disability Index 2.0; PCS, Pain Catastrophizing Scale; PSFS, Patient-Specific Functional Scale; QoL, quality of life; SES-SV, Self-efficacy Scale for Exercise; TSK, Tampa Scale for Kinesiophobia; SF-36, Short Form (36) Health Survey; VAS, Visual Analogue Scale of Pain

Data collection for the patient-reported outcome measures will take place at baseline (8–12 weeks before surgery), at 1 week before surgery; and at 3 weeks, 8 weeks, 3 months, 6 months, 1 year, 2 years and 5 years after surgery. Each participant will have a special numeric code, and all data will be stored in a fireproof cupboard and on a password-protected computer at one of the spine clinics. Double data entry and missing data in the questionnaires will be checked as soon as the participant has done the follow-up by an independent trial coordinator. Figure 4 presents details of the follow-up sessions.

### Analyses

#### Sample size

The number of participants (*n* = 110) was determined based on a power analysis (80 % power, alpha = 0.05), with disability measured with ODI as the primary outcome. We determined that we required a sample size of 55 patients in each group to show a statistically significant between-group difference of at least 8 points in the ODI with a standard deviation of 15 based on earlier studies [57, 63]. The difference of 8 points on the ODI was based on the previously reported minimal clinically important difference within the range of 5.2–16.3 where 10 points are mostly used for the ODI in a similar population [57, 64].

#### Effect evaluation

We will use an “intention to treat” approach to compare the effects of the different therapy conditions on both primary and all secondary outcomes. Data analysis will be performed using the statistics software SPSS 22 (IBM Corporation, New York, NY) for ODI at 6 months and all secondary outcomes at 6 months. The intervention group will be tested against the control group using two-sample *t*-test and Wilcoxon rank sum test depending on the data level of the outcomes. Interrelationship among the variables will be analysed using a multiple regression model with the ODI as outcome and the secondary outcomes as explanatory variables. In addition, possible mediations and interactions will be investigated. Since this trial will include repeated measures at different time-points, we will use a linear mixed model with random intercept [65].

To test the physical activity level between the groups at 3 and 6 months, we will use a two-sample *t*-test.

#### Health economics

For our health economic analysis, a societal viewpoint will be used. Indirect and direct healthcare costs will be measured using a self-reported custom-made health economic questionnaire. Days of hospital stay will be determined from patient charts. Intervention costs will be calculated for each patient – including the number and duration of treatments for the 55 patients who will receive PREPARE. We will compare the mean total costs between the therapy groups. The cost prices of medical consumption will be calculated. All costs will be presented in euros. The human capital approach will be used to calculate productivity costs.

We will use these data to perform a cost-effectiveness analysis and cost-utility analysis. For the cost-effectiveness analysis, an incremental cost-effectiveness ratio (ICER) will be calculated, weighing total costs against disability levels (ODI). For the cost-utility analysis, utility will be calculated from EQ-5D scores for every assessment. Mean total costs will be weighed against mean health utility, i.e. comparing cost per Quality Adjusted Life Years (QALY) gained.

## Discussion

The overall aim of our trial is to investigate whether patients experience improved post-operative functioning if they receive a pre-surgical physiotherapeutic prehabilitation program based on a cognitive behavioural approach, as compared to conventional care.

As stated previously, at the time that the present trial was designed, no prior investigation had examined a prehabilitation program with a cognitive behavioural approach. To this date, two studies have been published within the field [26, 30]. We would like to clarify the similarities and differences in order to argue for the added value of our planned trial, PREPARE. All three of the studies were set up with RCT designs, and disability was one of the primary outcomes [26]. Disability is however measured somewhat differently in the various studies. Nielsen et al. used the term functionality, which was assessed by the Roland Morris Disability Questionnaire and two performance-based outcome measures (the Sit-to-stand test and the Timed-up-and-go test) [26]. Rolving et al. used the ODI as the primary patient-reported outcome measure but did not use any performance-based outcome measures of functioning [30]. In PREPARE we use a combination of outcome measures (patient-reported and performance-based outcome measures as well as accelerometer data). We would argue that this will provide a more comprehensive picture of the patient’s functioning as reflected in the International Classification of Functioning, Disability and Health [66].

The intervention programs in the three studies mentioned above vary somewhat in the underlying theoretical models and content. PREPARE is being performed from a person-centred perspective [32]. In concrete terms, it means that we have set up person-centred treatment sessions on a one-to-one basis. This is in line with the trial from Nielsen et al. in which the healthcare professional met the patient on an individual basis before surgery. In their intervention program, the patient met with the physiotherapist on two occasions, at the day of inclusion and two weeks before surgery [26]. In order to gradually challenge the patient’s cognitive beliefs and to facilitate the patient’s self-efficacy in relation to staying active despite pain, we have included four sessions and one booster session.

As argued for in the introduction section, the main principle of our prehabilitation program is to help the patients to stay active despite pain. Both Nielsen et al. and Rolving et al. seem to have built their prehabilitation program on the same principle. Nielsen et al. investigated the effect of an individualised preoperative training program that focused on improvement of muscular back strength as well as cardiovascular conditioning before surgery [26]. Rolving et al., on the other hand, had a stronger cognitive behavioural focus of their intervention program [30]. The relevance of the pre-surgical psychological status is an important determinant of surgical outcome in PREPARE. In accordance with the modified fear-avoidance model, we hypothesise that by trying to influence catastrophizing thoughts and patient’s self-efficacy beliefs to exercise the patients in the intervention group will increase their level of functioning and physical activity level. In addition, in PREPARE we combine cognitive behavioural techniques with physical activity goal setting which we argue is a major strength that will further help the patients to be more active despite pain both before and after surgery.

### Trial status

Participant recruitment started in April 2014 and is planned to continue until December 2016. It is expected that data regarding the intervention effects and the economic analyses will be available at the end of 2017.

## Abbreviations

CLBP: Chronic Low Back Pain
DDD: Degenerative Disc Disease
EQ-5D: EuroQol 5 Dimensions Questionnaire
HADS: Hospital Anxiety and Depression Scale
ICER: Incremental Cost-Effectiveness Ratio
ODI: Oswestry Disability Index 2.0
PCC: Person-Centred Care
PCS: Pain Catastrophizing Scale
PSFS: Patient-Specific Functional Scale
QALY: Quality Adjusted Life Years
RCT: Randomised Controlled Trial
SEE-SV: Self-Efficacy Scale for Exercise
SF-36: Short Form (36) Health Survey
TSK: Tampa Scale for Kinesiophobia
VAS: Visual Analogue Scale
